# Clustering and disease subtyping in Neuroscience, toward better methodological adaptations

**DOI:** 10.3389/fncom.2023.1243092

**Published:** 2023-10-19

**Authors:** Konstantinos Poulakis, Eric Westman

**Affiliations:** ^1^Division of Clinical Geriatrics, Department of Neurobiology, Care Sciences and Society, Karolinska Institutet, Stockholm, Sweden; ^2^Department of Neuroimaging, Centre for Neuroimaging Sciences, Institute of Psychiatry, Psychology and Neuroscience, King's College London, London, United Kingdom

**Keywords:** clustering, unsupervised learning, Neuroscience, disease subtypes, Alzheimer's disease

The increasing interest in identifying disease biomarkers to understand psychiatric and neurological conditions has led to large patient registries and cohorts. Traditionally, clinically defined labels (e.g., disease vs. control group) were associated statistically with potential biomarkers to draw useful information about brain function related to a disease (supervised analysis) (Deo, 2015). However, the observed biomarker variability and the presence of clinical disease subtypes have sparked interest in quantitatively exploring heterogeneity (Feczko et al., 2019; Ferreira et al., 2020). The unsupervised^1^ exploration of a disease population (without any clinical labels) through a selected sample is a demanding task that differs from supervised analysis by definition (Habes et al., 2020). However, in research the differences between the two are often overlooked. Therefore, we want to highlight the applications and challenges of clustering, where supervised analysis principles are sometimes misapplied. We also demonstrate how such practices can negatively impact clustering results.

Some common challenges in clustering methods include selecting relevant features to describe data heterogeneity, preprocessing to remove biases, choosing appropriate similarity measures to summarize critical information, selecting a suitable method for meaningful clustering, tuning clustering model parameters (such as cluster size) without ground truth, and validating clustering results (Halkidi et al., 2001; Hennig et al., 2015).

The most common clustering applications in medicine (Halkidi et al., 2001):

- Data reduction (Hennig et al., 2015). When dealing with large datasets, like genomics, proteomics, or medical imaging data, clustering can condense the information into representative vectors or filter out uninformative features.
- Generate new hypotheses. Discovering specific disease subtypes can lead to the development of new hypotheses, altering existing theories.
- Hypothesis testing (Thrun and Ultsch, 2021). Clustering can be used for hypothesis testing. For example, it can assess whether clinical observations align with biological data in diseases with known subtypes without forcing the association between biological data and clinical labels (supervised approach).
- Prediction in new patients (Wu et al., 2019). Clustering can identify disease subtypes and scientific theories that investigators can use to create supervised classification models for grouping new patients. This new classification is valuable for personalized medicine and future patient treatment, among other applications.

When working with unsupervised methods, it's crucial to understand their limitations and nuances. Clustering encompasses a wide range of techniques which handle population structures and characteristics differently. Understanding the idiosyncrasies of a dataset is essential for applying clustering successfully. Questions about how clustering results generalize to the disease population, which are the optimal model parameters, and why results change with slight dataset modifications often emerge during study design, model optimization, interpretation, and peer review. One intriguing approach that combines automatic machine learning with expert knowledge from the field is the 'human-in-the-loop' method (Holzinger, 2016). This approach is particularly effective in neurological applications and can help address the abovementioned questions.

Regarding cluster size and type, we may know in advance whether there is excess variation in a disease population, some heterogeneous disease features, and even subtype proportions. This knowledge is vital in the model selection process so that we can sort out methods that are wrong methodological fits for the population of interest. For example, k-means, one of the most popular clustering methods, tends to produce convex-shaped clusters (it tends to equalize the spatial variance) that are spherical and often become similar in size (Celebi et al., 2013). Therefore, if in a specific disease population, we are aware of rare disease subtypes that may also exist in our sample, we may want to avoid k-means. Instead, we should focus on clustering methods to identify outliers/outlier clusters (Campello et al., 2015). Further, the more variables we use in a clustering method, the more the dimensionality of the dataset increases. A good practice is to use methods that either pretreat data to reduce the dimensionality and then apply regular clustering to them or select a method that can cope with high dimensional datasets (Babu et al., 2011; Thrun, 2021). While the gold standard in machine learning, some studies fail to utilize suitable models for high-dimensional data (Noh et al., 2014; Hwang et al., 2016; Jeon et al., 2019; Levin et al., 2021), limiting our ability to assess the success of clustering.

Further, all clustering methods cannot cope with all types of data (ordinal/nominal categorical, numerical) (Halkidi et al., 2001). When we binarize continuous variables to utilize a clustering algorithm for binary data only, the reduction of information due to data transformation must be at least considered when interpreting the results (Zhang et al., 2016). Some algorithms use mixed data types and should be preferred when mixed data distributions are present (Szepannek, 2019). If not accounted for, data biases may render a clustering result misleading. For example, we may be interested in understanding the heterogeneity of a particular biological process during aging. Understanding and adjusting the data to consider the participants' age variability results in clusters of participants that are not driven by age differences but by differences in the biological process under investigation if those exist (given that other biases are not present). However, due to complex data/aging relationships, these effects may persist even after statistical accounting for aging. Other sampling features that can drive clustering results are sex, disease stage, comorbidities, medication exposure, and geographical position. For example, it is known that the disease stage may contribute to the observed heterogeneity in Alzheimer's disease (AD) (Ferreira et al., 2020), we have only recently started accounting for this or trying to assess its contribution (Young et al., 2017; Vogel et al., 2021; Yang et al., 2021; Poulakis et al., 2022) while in previous studies (Noh et al., 2014; Dong et al., 2016; Hwang et al., 2016; Zhang et al., 2016; Park et al., 2017; Poulakis et al., 2018; ten Kate et al., 2018) we did not assess or account for this effect.

Clustering results must generalize well to the population, which makes validation a central topic. Traditionally, cross-validation (CV), bootstrapping, external data testing (training, validating, and testing), and careful sample selection have been some of the most popular approaches in supervised analysis. However, validation in clustering is not straightforward since no ground truth exists. The adaptation of training and testing a clustering model using independent datasets can sometimes mislead us. For example, three subtypes are present in a hypothetical disease population N (s1, s2, and s3). One is the most prevalent (s1) (typical presentation), the second subtype (s2) has half of the prevalence of the first one (n_s2_ = $\frac{1}{2}$n_s1_), and the third subtype has a low prevalence (one-tenth of the first subtype, n_s3_ = $\frac{1}{10}$n_s1_) (s3). The disease population N equals n_1_ + n_2_ + n_3_. A perfectly representative random sample of 100 patients from the disease population will include approximately 63 patients from s1, 31 from s2, and six from s3. A clustering model can then be trained on 70% (70 patients) and tested using 30% (30 patients). Suppose the data in the training set perfectly represent the population, a rare phenomenon, and clustering accurately identifies the subtypes. In that case, 44 patients will end up in Cluster 1, 22 in Cluster 2, and 4 in Cluster 3. The test set should have 19 patients in s1, 9 in s2, and 2 in s3. Clustering can then be applied to identify subtypes s1, s2, and s3. Since the actual data labels are unknown, which is what clustering should discover, the test set results will be compared to the training set. The problem arises with rare subtypes, such as the hypothetical s3 subtype (six patients in the sample, four in the training set, and two in the test set). Patients of such subtypes may end up in larger clusters when the overall dataset is split into small segments for the needs of the analysis. Unfortunately, the most interesting heterogeneous characteristics will enrich another cluster's greater information pool, especially in high-dimensional datasets. In the best-case scenario, those patients will be single outliers (if the algorithm can recognize outlier clusters) (Campello et al., 2015). Understanding their features is pivotal for the assessment of heterogeneity in the disease.

To the best of our knowledge, cross-validation has been successfully combined with clustering in two studies to assess the consistency of observations within the same cluster and to determine the optimal model solution (Varol et al., 2017; Yang et al., 2021). On the other hand, leave 10% of patients out-CV (a semi-supervised application where a control group is contrasted to a disease group) to decide the optimal clustering (Dong et al., 2016, 2017), may reveal the dominant patterns in the dataset. An interesting question is whether clusters of low/very low prevalence can survive this process. In AD, genetic mutations account for <1% of all AD (2020) cases, while early-onset AD accounts for 4%−6% (Mendez, 2017). Another evaluation approach is to compare clustering agreement after application of the same algorithm in different cohorts. We do not suggest that these results are wrong, but they may be misleading if different clustering findings in different cohorts are interpreted as a methodological failure, while convergence of findings between cohorts is the aim (ten Kate et al., 2018; Vogel et al., 2021). Sometimes, it is a requirement that clustering should be repeated cohort-wise to prove model robustness (Poulakis et al., 2018, 2022). Instead of reducing data variability in clustering by splitting the available data into segments, we should acknowledge that cluster-cohort agreement-based evaluation criteria can potentially interrupt the discovery of rare data patterns. Another issue with the cohort-wise analysis is the potential sample imbalance between cohorts that may render one cohort solution less reliable than another. Of note, cohort-wise analysis is reasonable when cohorts have different feature sets or systematic differences (Marinescu et al., 2019; Tijms et al., 2020). Prior knowledge (subtype prevalence or number of subtypes) is essential when formulating a clustering experimental design (Halkidi et al., 2001, 2002). Another example, hypothetically, two separate clusters of patients may be formed because a clustering validation criterion gives marginally better scores instead of grouping the patients in one cluster. Field experts and not only clustering internal evaluation criteria should conclude whether differences between clusters are essential enough to suggest heterogeneity (Halkidi et al., 2002; Dolnicar and Leisch, 2010). It is also often observed that clustering algorithms optimally select two-cluster solutions. This finding may not provide any insight of the disease process when it only reveals biomarker severity differences of no clinical interest (Poulakis et al., 2021; Yang et al., 2021). Based on the above, we believe that as large datasets as possible should be used when training a clustering model. In contrast, datasets should not be divided for validation purposes if the focus is on revealing heterogeneity in a population.

Clustering is a valuable approach to understand heterogeneity in brain disorders and healthy aging. The machine learning community has invested a great deal of research in addressing the methodological issues discussed above. As with every statistical tool, these methods should be carefully applied, and understanding their properties and limitations is essential.

## Author contributions

All authors listed have made a substantial, direct, and intellectual contribution to the work and approved it for publication.

## Footnotes

^1^For the needs of this text, unsupervised analysis refers to clustering only, association analysis is not covered.
